# Proximity‐sensors on GPS collars reveal fine‐scale predator–prey behavior during a predation event: A case study from Scandinavia

**DOI:** 10.1002/ece3.10750

**Published:** 2023-12-11

**Authors:** Aimee Tallian, Jenny Mattisson, Fredrik Stenbacka, Wiebke Neumann, Anders Johansson, Ole Gunnar Støen, Jonas Kindberg

**Affiliations:** ^1^ Norwegian Institute for Nature Research Trondheim Norway; ^2^ Department of Wildlife, Fish, and Environmental Studies Swedish University of Agricultural Sciences Umeå Sweden; ^3^ Sveaskog Sveg Sweden; ^4^ Norwegian Institute for Nature Research Oslo Norway

**Keywords:** *Alces alces*, behavioral interactions, brown bears, direct interactions, fine‐scale movement, interspecific interactions, moose, predator–prey interactions, Sweden, *Ursus arctos*

## Abstract

Although the advent of high‐resolution GPS tracking technology has helped increase our understanding of individual and multispecies behavior in wildlife systems, detecting and recording direct interactions between free‐ranging animals remains difficult. In 2023, we deployed GPS collars equipped with proximity sensors (GPS proximity collars) on brown bears (*Ursus arctos*) and moose (*Alces alces*) as part of a multispecies interaction study in central Sweden. On 6 June, 2023, a collar on an adult female moose and a collar on an adult male bear triggered each other's UHF signal and started collecting fine‐scale GPS positioning data. The moose collar collected positions every 2 min for 89 min, and the bear collar collected positions every 1 min for 41 min. On 8 June, field personnel visited the site and found a female neonate moose carcass with clear indications of bear bite marks on the head and neck. During the predation event, the bear remained at the carcass while the moose moved back and forth, moving toward the carcass site about five times. The moose was observed via drone with two calves on 24 May and with only one remaining calf on 9 June. This case study describes, to the best of our knowledge, the first instance of a predation event between two free ranging, wild species recorded by GPS proximity collars. Both collars successfully triggered and switched to finer‐scaled GPS fix rates when the individuals were in close proximity, producing detailed movement data for both predator and prey during and after a predation event. We suggest that, combined with standard field methodology, GPS proximity collars placed on free‐ranging animals offer the ability for researchers to observe direct interactions between multiple individuals and species in the wild without the need for direct visual observation.

## Graphical abstract

We deployed GPS collars equipped with proximity sensors (GPS proximity collars) on brown bears and moose as part of a multispecies interaction study in central Sweden. On 6 June, 2023, a marked bear preyed on the calf of a marked moose, and both collars successfully triggered and switched to finer‐scaled GPS fix rates when the individuals were in close proximity, producing detailed movement data for both predator and prey during and after a predation event. This case study describes, to the best of our knowledge, the first instance of a predation event between two free‐ranging, wild species recorded by GPS proximity collars.

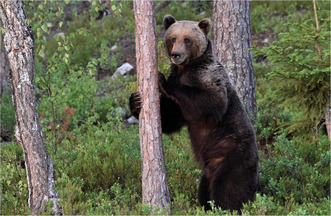

## 1 INTRODUCTION

Understanding how animals interact with one another is a fundamental goal of ecology. Although studying interactions between free‐ranging animals is challenging, the advent of high‐resolution GPS tracking technology has helped increase our understanding of multi‐individual and multispecies behavior in wild systems (Hussey et al., 2015; Kays et al., 2015; Williams et al., 2020). GPS technology has proven especially useful for exploring predator–prey interactions in natural systems (Kays et al., 2015). For example, deploying GPS collars on free‐ranging large mammals has helped investigate and quantify multiple aspects of the predator–prey relationship, including predator and prey movement and habitat selection, prey antipredator behavior and foraging strategies, and the overall impact of predators on prey populations (Hebblewhite & Haydon, 2010; Kays et al., 2015; Wilmers et al., 2015). However, detecting and recording direct interactions between free‐ranging individuals remains difficult.

Despite the rarity of witnessing encounters in the wild, much of what we know about the outcome of direct interactions between individuals and what current ecological theory is generally based on comes from observational studies (e.g., Cassidy et al., 2017; MacNulty et al., 2014; Zarzo‐Arias et al., 2018) or chance observations (e.g., Laidre et al., 2006; MacNulty et al., 2001). Observational studies can be successfully implemented in systems with optimal conditions; for example, Yellowstone's landmark long‐term observational study on wolves was conducted in an area with open terrain and abundant hilltop viewpoints (Smith et al., 2020). Yet, most study systems do not offer such ease of access for wildlife viewing; for example, many are characterized by a combination of closed, forested terrain, few vantage points, remote areas, and cryptic or shy species. Current technological advances in GPS collar capabilities, however, offer a unique opportunity to detect and record direct interactions between individuals in the wild without the need for direct observation (Wilmers et al., 2015).

Proximity sensors for GPS collars are a novel technology that allow for detailed study of focal individuals (Græsli et al., 2020; Le Grand et al., 2019; Støen et al., 2022). Proximity sensors both transmit and detect weak ultra‐high‐frequency (UHF) signals. When a UFH signal is detected, the GPS collar will “trigger” and switch to an alternate fix rate for a pre‐defined time interval. In other words, collars normally set to a coarse fix rate (e.g., 1 h) can switch to a finer‐scale fix rate (e.g., 1 min) for a specified time period when they come in the “proximity” of another UHF transmitter device. Proximity sensors therefore offer the possibility to switch GPS collars to collect fine‐scale GPS data during specific events and then switch back to coarser fix rates once the event is over, saving battery life, extending collar life, and delaying invasive, time‐intensive, and costly recapture events.

Collars equipped with proximity triggers have already been deployed in the field to explore human–wildlife interactions (Græsli et al., 2020; Le Grand et al., 2019) and assess predation patterns in carnivore‐ungulate systems (Støen et al., 2022). For example, Støen et al. (2022) evaluated brown bear (*Ursus arctos*) predation on semi‐domestic reindeer (*Rangifer tarandus tarandus*) in Scandinavia by equipping brown bears with GPS proximity collars and reindeer with simple neck collars that carried UHF transmitters; that is, reindeer collars did not have GPS capability. In this experiment, bear collars collected fine‐scale positional data when they came in close proximity to reindeer, while the passive reindeer collars collected no data. To the best of our knowledge, GPS proximity collars have yet to be simultaneously deployed on two free‐ranging species to detect interspecific interaction events and record fine‐scale movement in the wild.

We deployed GPS collars on brown bears and moose (*Alces alces*) and followed them during spring (10 May–25 June) 2023 in Sweden as part of a multispecies interaction study. The primary goal of the study was to evaluate bear‐moose kill rates via an on‐the‐ground predation study. This timeframe spanned the moose parturition period from mid‐May until mid‐June (Neumann et al., 2020), which is also the primary bear‐neonate moose predation period in Sweden (Rauset et al., 2012; Swenson et al., 2007). We also equipped a subsample of moose and bear GPS collars with proximity sensors, with the secondary goal of exploring their capacity to opportunistically collect fine‐scale movement data during direct interactions between brown bears and moose.

## 2 MATERIALS AND METHODS

### 2.1 Study area

**FIGURE 1:**
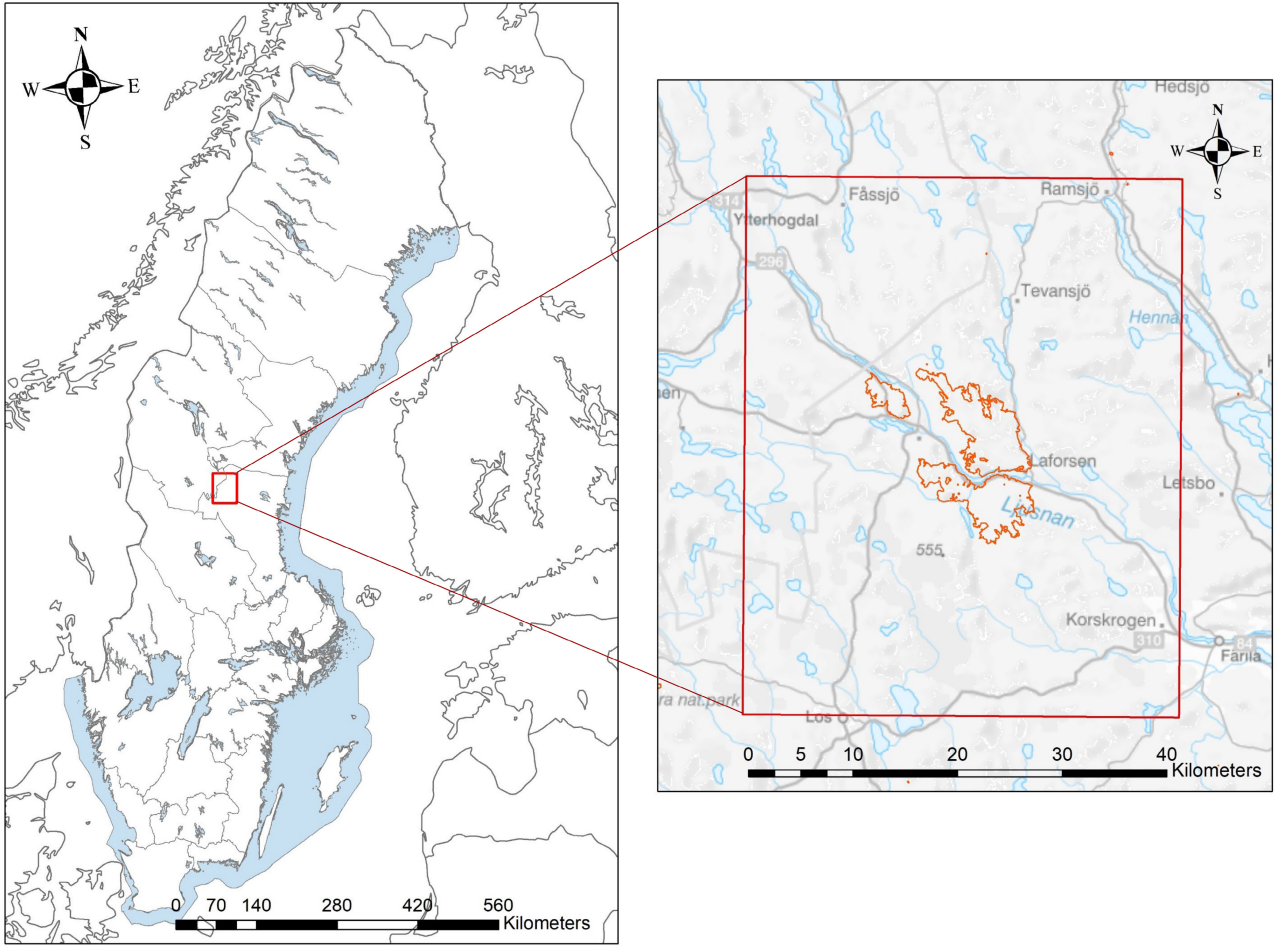
Map of the study area in central Sweden (red box). The burned area from 2018 is highlighted in orange.

Our study was conducted in an area in central Sweden (~2500 km^2^, elevation ~50–600 m) in the Ljusdal and Härjedalen Municipalities of Gävleborg and Jämtland Counties, respectively (Figure 1). The rolling landscape is mostly comprised of intensely managed boreal forest, which is dominated by Scots pine (*Pinus sylvestris*) and Norway spruce (*Picea abies*). The understory is dominated by heather, berry‐producing shrubs, and grasses. In 2018, the Ljusdal municipality experienced multiple forest fires that burned approximately 8400 hectares (84 km^2^) of forest near the center of the study area (Figure 1).

The Scandinavian brown bear population was estimated at ~3300 individuals in 2008 and ~2750 individuals in 2018, with densities reaching three bears per 100 km^2^ (Bischof et al., 2020; Kindberg et al., 2011). Bears in Scandinavia use a wide variety of plant and animal foods throughout the year (Stenset et al., 2016) and prey on neonate moose during early summer (i.e., May–June; Rauset et al., 2012), but rarely kill adult ungulates (Dahle et al., 2013). Alternative ungulate prey in the area included red deer (*Cervus elaphus*). Other predators in the area included wolves (*Canis lupus*), wolverines (*Gulo gulo*), and golden eagles (*Aquila chrysaetos*).

The moose density in the area is estimated at 4–9 moose per 10 km^2^. Moose commonly reproduce annually between May and June (Neumann et al., 2020; Niedziałkowska et al., 2022); the mean birthing date for the study area is 18 May (Neumann et al., 2023). For moose, the twinning rate is closely related to female age and habitat quality, reaching a maximum level in prime‐aged females (Sæther & Haagenrud, 1983). Within the study area, 63% of calves born to GPS‐marked females are born as twins. For adult moose, human harvest is the main source of mortality, even in areas with large predators, whereas predators can reduce calf survival considerably (Niedziałkowska et al., 2022; Sand et al., 2006; Sivertsen et al., 2012; Swenson et al., 2007).

### 2.2 Capturing and collaring

Bears and female moose were captured and collared via helicopter using established protocols (Arnemo et al., 2012; Kreeger & Arnemo, 2007; Lian et al., 2014), which were approved by the Swedish Ethical Committee on Animal Research; Permits Dnr 5.8.18‐03376/2020 and Dnr A11‐2020. Moose capture efforts began in 2020, with the goal of collaring females near the 2018 burn and within the core study area (Figure 1). Bear capture efforts began in 2022 and were focused on the area where moose had previously been collared to maximize temporal and spatial overlap between species and, thus, the potential to observe interspecific interactions.

Captured bears and moose were equipped with GPS neck collars (Vectronic Aerospace). During the 2023 capture, a subsample of bears (*n* = 4; two adult males, one solitary female, and one female with cubs of the year) and moose (*n* = 18) were fitted with GPS neck collars that also had proximity sensors and UHF transmitters, that is, GPS proximity collars (Vectronic Aerospace GmbH, Berlin, Germany). Proximity collars are equipped with a UHF transmitter and receiver; the transmitter sends a weak UHF signal while the receiver scans for other UHF signals (see Table 1 for detailed settings). Once a signal was received by a collar, the collar reconfigured to a pre‐determined fixed schedule and logged the ID of the collar that was triggered by it. Once the signal was lost, the collar reverted to its original programming after a pre‐scheduled amount of time. The range of UHF signal detection is based on terrain and cover but is usually about 100 m or so away.

**TABLE 1:** Collar and proximity function settings for the bear and moose collars during the 2023 study season.

GPS proximity collar settings		
Active proximity period: 10 March to 25 June	Bears	Moose
UHF transmitter settings		
Beacon frequency	443,000	443,000
Beacon power	10 dBm	10 dBm
Beacon pulse length	5 ms	5 ms
Beacon loop length	1250 ms	1250 ms
UHF receiver settings		
Receiver sensitivity		
Listen duration	1.5 s	1.5 s
Listen interval	2 min	5 min
Start/end time	24 h	24 h
ID Blacklist	All bear collars	All moose collars
ID Whitelist	None	None
Skip count	No	No
Sample count	Not applicable	Not applicable
Triggered collar settings		
Fix rate	1 min	2 min
Fix duration	15 min	60 min

*Note*: The active proximity period defines the timeframe where the proximity function is active on the collars. UHF transmitter settings include the frequency (MHz) and power (dBm) of the UHF beacon and the pulse length and loop length (cycle in which the signal is repeated) in milliseconds (ms). UHF receiver settings include the receiver sensitivity (dBm), the listen duration and interval (e.g., the collar listens for 2000 ms (2 s) every 5 min), the start/end time (the time of day the collar listens), ID blacklist (a list of collar IDs that can be ignored if the signal is received), ID whitelist (a list of specific collar IDs that will trigger the proximity settings; if undefined, it triggers on all except the ID blacklist), skip count (how many GPS positions from a proximity event will be uploaded versus stored on board; no skip count means all GPS positions will be uploaded), and sample count (if a skip count, how many proximity data are stored on board). The pre‐defined GPS fix rates and durations (e.g., the collars take GPS positions every minute for 15 min) are implemented when another UHF signal is received and are active until the signal is lost.

Bear proximity collars were programmed to take GPS positions every 30 min and increase to a fixed rate of 1 position every 1 min for a duration of 15 min when they came within range of another UHF signal (Table 1). Moose proximity collars were programmed to take GPS positions every 30 min and increase to a fixed rate of 1 position every 2 min for a duration of 60 min when they came within range of a proximity‐collared bear (Table 1); the 2‐min setting was chosen to save battery life over the longer fix duration. Using the GSM‐network or IRIDIUM satellite, the collars send continuously positions to the existing database Wireless Remote Animal Monitoring (Dettki et al., 2014) at the Swedish University of Agricultural Sciences, which allows us to monitor animals remotely in near real time.

### 2.3 General study design

The overarching goal of the project was to conduct a field predation study during the moose parturition period to quantify bear‐moose kill rates. GPS data were downloaded daily and used to generate “GPS clusters” that were subsequently visited by field crews. GPS clusters were defined as ≥2 overlapping positions within a 30 m radius of one another (Rauset et al., 2012). Between 10 May and 25 June, 2023, field crews searched clusters within a 50 ‐m radius for prey remains optimally no later than 3 days after they were generated, or sooner if the bear left the area. For each prey remain, field crews identified the species, age, sex, cause of death (i.e., focal bear, other bear, other predator, non‐predator), and time of death (first GPS point of the focal bear within the cluster). Between 16 May and 14 June, 2023, we confirmed the number of calves born to GPS‐collared female moose and their status (i.e., alive or dead) through field observations either on foot or via drone (DJI Mini 2). These “calf checks” were performed by identifying changes in females' movement patterns using the GPS data (i.e., calving clusters), which suggested that females had given birth (Neumann et al., 2020; Nicholson et al., 2019). A secondary goal of the study was to explore the capacity of GPS proximity collars to detect and document interspecific interactions and collect fine‐scale movement data in the wild. Such data are collected opportunistically (i.e., when two study animals come close enough to detect each other's UHF signals and trigger their collars).

## 3 RESULTS

On 24 May, 2023, field crews recorded moose F4692 (adult female) via drone at her calving site with two newborn calves (Video 1) at 14:50 local time. Both calves appeared to be healthy and able to stand at the time the observation was recorded. Their date of birth was estimated to be 23 May, 2023 after 18:00 (first GPS position in the cluster).

**VIDEO 1:** Drone footage of the adult female moose F4692 was taken via drone at 14:50 local time on 24 May, 2023. The video shows moose F4692 and her two newborn calves at the calving site (the site where she gave birth). The site is located on a small island with pine forests surrounded by a bog and open water. The video is credited to Anders Johansson.

**FIGURE 2:**
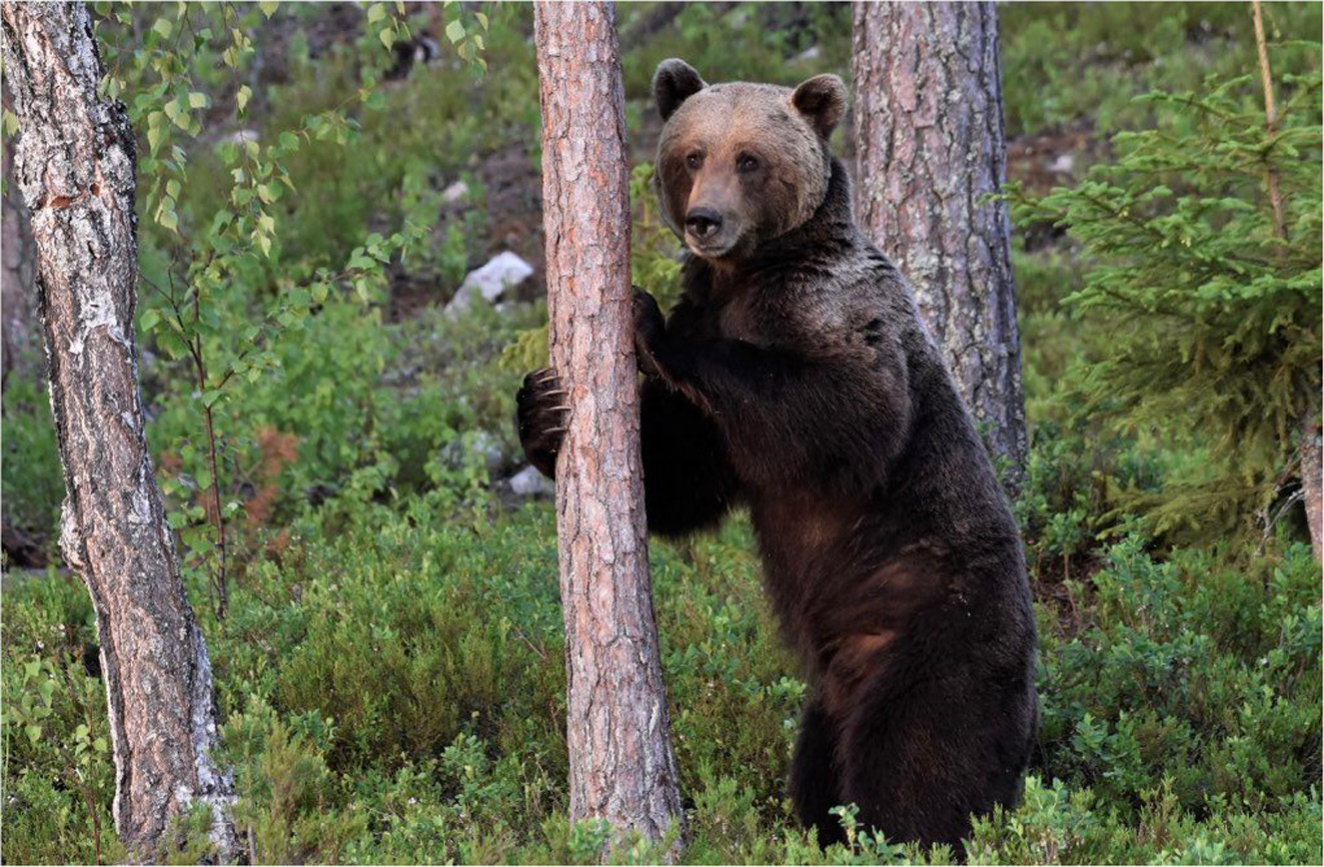
Bear W2306, standing near a tree. Photo taken by a trail camera in another part of the study area. Photo credited to Marco Hassold/Wildlife Sweden.

On 6 June, 2023, the collars on moose F4692 and bear W2306 (adult male; Figure 2) both triggered on each other's UHF signal and started collecting fine‐scale GPS positioning data. Bear W2306's collar triggered at 18:30 local time at a distance of 107 m to the moose and collected 1‐min positions for the next 41 min, ending at 19:10. The collar returned to normal functioning at 19:30 local time. Moose F4692's collar triggered at 18:28 local time and collected 2‐min positions for the next 89 min, ending at 19:56. The collar returned to normal functioning at 20:00 local time.

**FIGURE 3:**
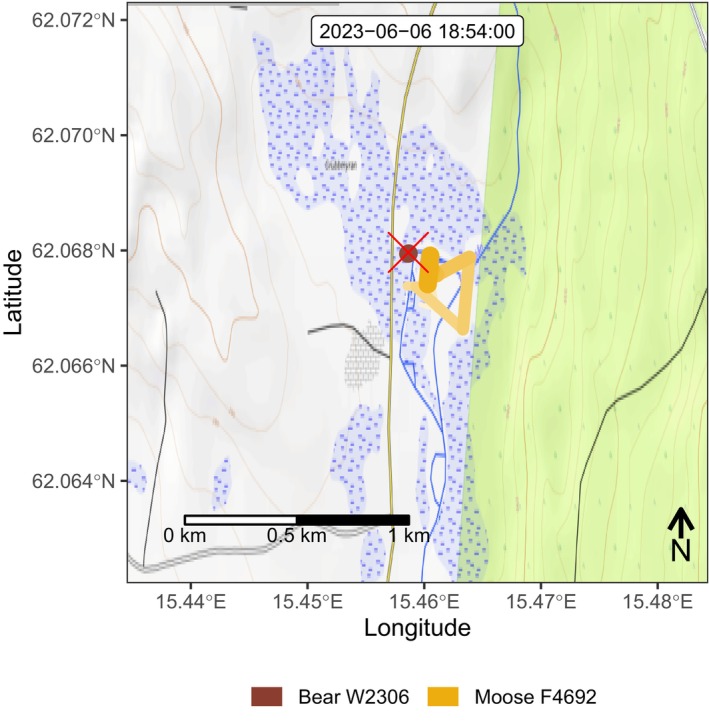
A map of the area where the proximity event occurred. Bear W2306's movement path is in brown and moose F4692's in yellow. The calf carcass location is marked by a red X.

Bear W2306 moved very close to moose F4692 at her calving site at 18:30, presumably either capturing one of the newborn calves quickly and then moving across the river to eat it or chasing into or across the river to capture it (Figure 3, Video 2). GPS data indicate that moose F4692 then moved back and forth in the area of the predation event for the next ~65 min within a radius of about 600 m off the predation site, repeatedly moving toward the calf predation site (about five different times) and then away from it again. The closest she came to the bear in a recorded position was 44 m at 19:24, but she may have moved closer between GPS fixes. Bear W2306 and moose F4692 both moved away from the site at ~19:30.

**VIDEO 2:** Animation depicting the locations of moose F4692 and bear W2306 in real time in relation to one another, the calving site, and the predation site. Moose F4692 comes as close as 40 m to the predation site at 19:24 local time.

**FIGURE 4:**
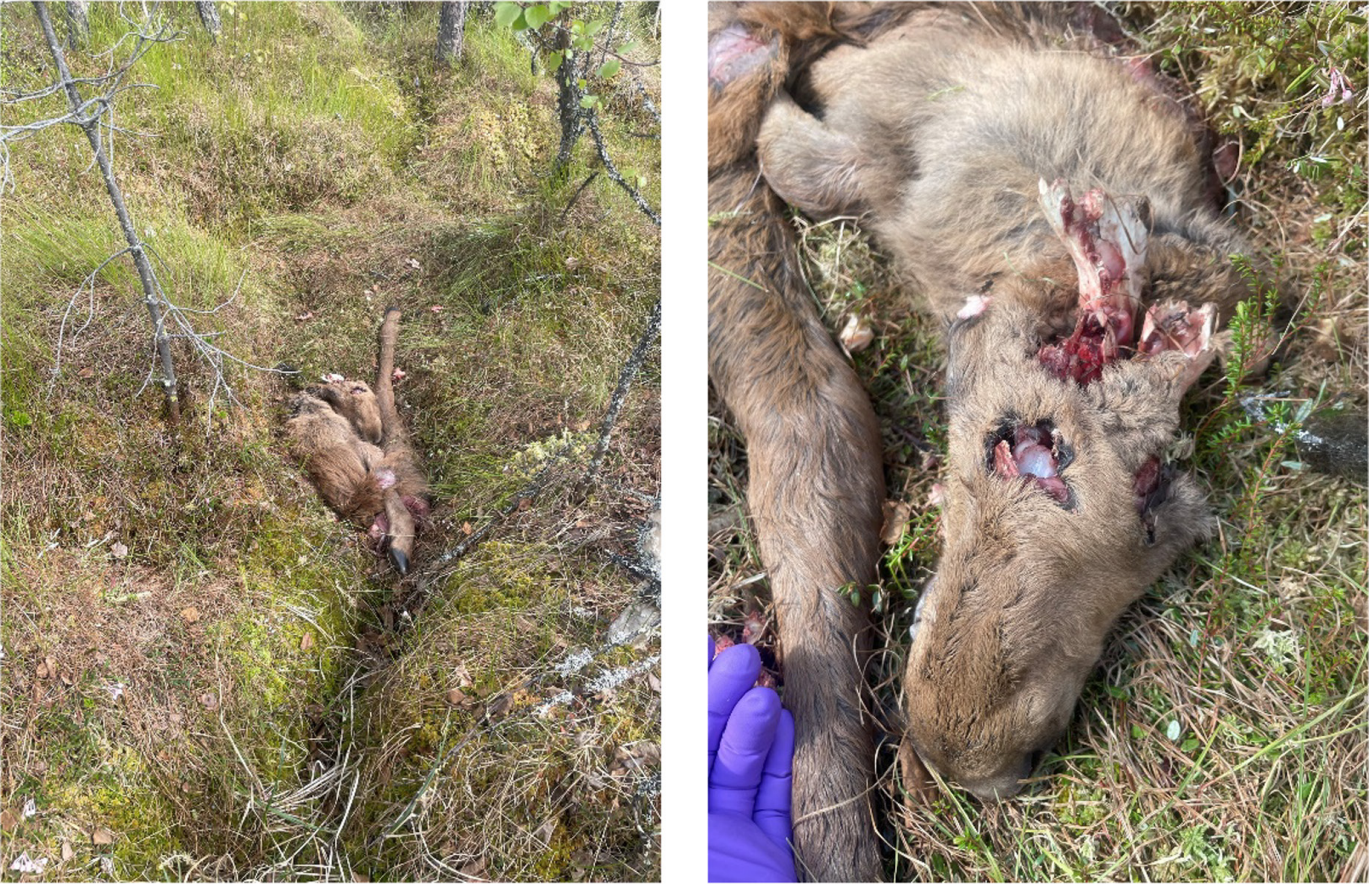
The predation site and remains of moose F4692's female calf. Photos credited to Jenny Mattisson.

On 8 June, field crew visited the site and found a female neonate moose carcass with clear indications of bear bite marks on the head and neck (Figure 4). The calf carcass was located in a bog/mire area and was approximately 60% consumed (Figure 4). The time of death was estimated to be 18:30 local time (based on the bear's GPS locations), and the calf's age at death was 14 days.

On 9 June, 2023, field crews observed moose F4692 via drone approximately 800 m away from the predation site with one remaining calf of the year at 10:45 local time (Video 3).

**VIDEO 3:** Drone footage of the adult female moose F4692 taken via drone at 10:45 local time on 9 June, 2023. The video shows the one remaining calf of moose F4692 in a mixed forest/bog area. The footage was taken approximately 800 m away from the predation site. The video is credited to Anders Johansson.

## 4 DISCUSSION

We have described, to the best of our knowledge, the first instance of a predation event between two free‐ranging wild predators and prey species recorded by GPS proximity collars. Both collars successfully triggered and switched to fine‐scaled GPS fix rates when the individuals were in close proximity, producing detailed movement data for both predator and prey during and after a predation event. That we only recorded one interaction that resulted in a predation event during a ~2‐month study period reinforces the opportunistic nature of the data collection effort. It also highlights the need to deploy proximity collars over the course of long‐term studies in order to build direct‐interaction datasets robust enough to undergo rigorous statistical analysis.

Combined with standard field methodology, GPS proximity collars placed on free‐ranging animals offer researchers the ability to explore interactions remotely and without disturbance and potentially detect novel behaviors. In our study, fine‐scale movement data of female moose during and after a predation event helped shed light on how moose behave in the moments after one of their calves has been killed and the risks they are willing to take to defend or check on their calf. Neonate ungulate calves are highly vulnerable to predation by bears (Griffin et al., 2011; Swenson et al., 2007), and a female moose with a calf at‐heel that remains close to a bear during a predation event could increase the risk of predation for her other calf. Research generally suggests moose behave in a way that reduces predation risk for both them and their calves, often trading off foraging for safety (Montgomery et al., 2013; Pusenius et al., 2020). However, the observation of moose F4692 on 9 June showed she still had one living calf during the predation event and that she stayed in the vicinity of the bear for some time after the first calf was taken. This suggests female moose may remain near a predation site to defend or check on their downed calf, even at the cost of increased acute risk for the second calf.

GPS proximity collars also have the potential to help refine ongoing field methodology and practices. For example, bear W2306 stayed at the moose calf carcass for <45 min. The main predation study used GPS clusters to detect bear‐moose predation events, which were defined as ≥2 overlapping 30‐min positions within a 30 m radius of one another (Rauset et al., 2012). The recorded proximity event suggested that the range of neonate moose handling times may sometimes be shorter than expected, which could result in undetected neonate moose bear kills and, ultimately, skewed estimates of bear kill rates. To estimate the bear kill rate as accurately as possible, the ongoing predation study will hereafter define GPS clusters as ≥2 overlapping 15‐min positions within a 30 m radius of one another.

The limitations of both this study and the overall use of GPS proximity collars on free‐ranging animals to detect and record direct interactions are relatively straightforward. First, the opportunistic nature of the data collection implies that collecting a large enough sample to robustly evaluate behavioral interactions will take time. However, deploying GPS proximity collars concurrently in field studies with other research objectives presents an opportunity to build a unique dataset that can answer questions about how free‐ranging species interact with one another in environments that inhibit direct observations (e.g., during night, in forests, in very remote places). Thus, we suggest that GPS proximity deployments to evaluate behavioral interactions on wild animals might not be the main focus of any given study but rather an opportunistic addition. Second, it is well understood that GPS proximity collars cannot yet be deployed on all free‐ranging species; for example, given current technology, only ~35% of all terrestrial mammals are large enough to even be equipped with tags that transmit GPS data to users in real time (Kays et al., 2015). Yet the battery demands for the fine‐scale fix interval, even when restricted to short events, further limit the deployment of GPS‐equipped proximity collars to relatively larger‐bodied mammals. However, this suggests that large mammals represent a unique opportunity to utilize GPS proximity collars to study behavioral interactions in the wild, which can be valuable or even crucial data for the management of multispecies systems (e.g., predator–prey or ungulate communities).

Despite the limitations, we suggest this technology can be used to explore a wide range of individual and multispecies interactions between large mammals in free‐living systems. As shown by this case study, proximity collars have the potential to offer valuable insight into the behavior of wild predators and their prey. For example, recent debate over the effect of wolves on elk (*Cervus elaphus*) highlights the need to match the temporal scale of GPS data collection with the behavioral interaction that is being studied (Kays et al., 2015); a study by Middleton et al. (2013) found wolves had little effect on elk behavior as actual wolf–elk encounters, detected via GPS collars, were rare, while Creel et al. (2013) rebutted that observed encounters might be rare because those events were short in duration and thus unable to be detected by the coarser GPS fix rate used in the study. In the aforementioned scenario, the deployment of proximity collars on both predator and prey species would have provided a better estimate of direct interactions.

It is important to note that the use of this technology is not limited to predator–prey interactions but can be used to evaluate a wide array of inter‐specific interactions, including interactions between humans and wildlife. For example, GPS proximity collars could be deployed to explore the outcome of direct competitive inter‐ or intraspecific interactions for any larger species. Examples include exploring direct interactions between predators at kill sites or agonistic interactions between competing groups or individuals. Researchers might also use GPS proximity collars to explore species' reproductive and mating behaviors. However, caution is required when deploying proximity collars, as primary data collection objectives must be balanced with the decreased collar life associated with proximity events.

## AUTHOR CONTRIBUTIONS

**Aimee Tallian:** Conceptualization (lead); data curation (supporting); formal analysis (lead); funding acquisition (equal); investigation (equal); methodology (lead); visualization (lead); writing – original draft (lead); writing – review and editing (lead). **Jenny Mattisson:** Conceptualization (equal); data curation (equal); formal analysis (supporting); funding acquisition (supporting); investigation (equal); methodology (equal); visualization (supporting); writing – original draft (supporting); writing – review and editing (equal). **Fredrik Stenbacka:** Conceptualization (equal); data curation (equal); formal analysis (supporting); funding acquisition (equal); investigation (equal); methodology (equal); writing – review and editing (supporting). **Wiebke Neumann:** Conceptualization (equal); data curation (equal); formal analysis (supporting); funding acquisition (equal); investigation (equal); methodology (equal); project administration (lead); writing – review and editing (supporting). **Anders Johansson:** Data curation (supporting); funding acquisition (supporting); investigation (supporting); resources (supporting); visualization (supporting); writing – review and editing (supporting). **Ole Gunnar Støen:** Conceptualization (equal); funding acquisition (supporting); investigation (supporting); methodology (equal); writing – original draft (supporting); writing – review and editing (supporting). **Jonas Kindberg:** Conceptualization (equal); funding acquisition (lead); investigation (equal); methodology (supporting); project administration (lead); supervision (lead); writing – original draft (supporting); writing – review and editing (equal).

## CONFLICT OF INTEREST STATEMENT

We have no competing interests.

## Data Availability

Data are available at Tallian et al. (2023). Dryad: https://doi.org/10.5061/dryad.pc866t1wf.
